# Supplementary material for: Methods for Fungicide Efficacy Screenings: Multiwell Testing Procedures for the Oomycetes Phytophthora infestans and Pythium ultimum
Source: Microorganisms. 2023 Jan 31;11(2):350. doi: 10.3390/microorganisms11020350 (PMC9959339; doi:10.3390/microorganisms11020350)
Supplement: Supplementary file 1 [file microorganisms-11-00350-s001.zip › microorganisms-2112056-supplementary.pdf]

Table S1: BRM estimated coefficients for each variable ($X_j$ = assay type; $X_i$ = mandipropamid concentration). Standard error (SE) and p-values from t-statistics are also reported.

| *Coefficient* | *Estimate* | *SE* |
|---|---|---|
| *Model's intercept ($\beta_0$)* | -2.91*** | 0.26 |
| *$X_j$ (96-wells)* | -0.42 (.) | 0.23 |
| *$X_j$ (9 cm Petri)* | -0.49* | 0.22 |
| *$X_i$ (1 ng/l)* | -0.21 | 0.31 |
| *$X_i$ (10 ng/l)* | 4.83*** | 0.28 |
| *$X_i$ (100 ng/l)* | 6.94*** | 0.36 |
| *$X_i$ (1000 ng/l)* | 6.92*** | 0.36 |

$X_j$ (24-wells) = 0; $X_i$ (0.1 ng/l) = 0

Model's evaluation: Pseudo $R^2$: 0.95; Root mean square error (RMSE): 0.044

P-values = 0 '***' 0.001 '**' 0.01 '*' 0.05 '(.)' 0.1 ' ' 1

Table S2: EMMs (back-transformed to percentages) obtained from the BRM for each assay (9 cm Petri, 24-wells and 96-wells) at each mandipropamid concentration. Confidence interval limits (95%) are reported in brackets.

| *mandipropamid concentration (ng/l)* | *9 cm Petri* | *24-wells* | *96-wells* |
|---|---|---|---|

| | | | |
|---|---|---|---|
| *0.1* | 3.21 (1.66 - 4.75) | 5.14 (2.69 - 7.6) | 3.43 (1.7 - 5.16) |
| *1* | 2.62 (1.29 - 3.95) | 4.22 (2.09 - 6.34) | 2.8 (1.32 - 4.28) |
| *10* | 80.55 (74.47 - 86.62) | 87.14 (82.65 - 91.64) | 81.6 (76 - 87.21) |
| *100* | 97.15 (95.68 - 98.62) | 98.24 (97.23 - 99.25) | 97.33 (95.88 - 98.79) |
| *1000* | 97.11 (95.62 - 98.59) | 98.21 (97.19 - 99.23) | 97.29 (95.82 - 98.77) |
